# Pregnancy-associated changes in urinary uromodulin excretion in chronic hypertension

**DOI:** 10.1007/s40620-023-01830-6

**Published:** 2024-01-18

**Authors:** Sheon Mary, Fran Conti-Ramsden, Philipp Boder, Humaira Parveen, Dellaneira Setjiadi, Jessica Fleminger, Anna Brockbank, Delyth Graham, Kate Bramham, Lucy Charlotte Chappell, Christian Delles

**Affiliations:** 1 School of Cardiovascular and Metabolic Health, BHF Glasgow Cardiovascular Research Centre, University of Glasgow, 126 University Place, Glasgow, G12 8TA UK; 2 Department of Women and Children’s Health, King’s College London, London, UK

**Keywords:** Chronic hypertensive pregnancy, Kidney physiology, Uromodulin, Blood pressure

## Abstract

**Background:**

Pregnancy involves major adaptations in renal haemodynamics, tubular, and endocrine functions. Hypertensive disorders of pregnancy are a leading cause of maternal mortality and morbidity. Uromodulin is a nephron-derived protein that is associated with hypertension and kidney diseases. Here we study the role of urinary uromodulin excretion in hypertensive pregnancy.

**Methods:**

Urinary uromodulin was measured by ELISA in 146 pregnant women with treated chronic hypertension (*n* = 118) and controls (*n* = 28). We studied non-pregnant and pregnant Wistar Kyoto and Stroke Prone Spontaneously Hypertensive rats (*n* = 8/strain), among which a group of pregnant Stroke–Prone Spontaneously Hypertensive rats was treated with either nifedipine (*n* = 7) or propranolol (*n* = 8).

**Results:**

In pregnant women, diagnosis of chronic hypertension, increased maternal body mass index, Black maternal ethnicity and elevated systolic blood pressure at the first antenatal visit were significantly associated with a lower urinary uromodulin-to-creatinine ratio. In rodents, pre-pregnancy urinary uromodulin excretion was twofold lower in Stroke-Prone Spontaneously Hypertensive rats than in Wistar Kyoto rats. During pregnancy, the urinary uromodulin excretion rate gradually decreased in Wistar Kyoto rats (a twofold decrease), whereas a 1.5-fold increase was observed in Stroke-Prone Spontaneously Hypertensive rats compared to pre-pregnancy levels. Changes in uromodulin were attributed by kidney injury in pregnant rats. Neither antihypertensive changed urinary uromodulin excretion rate in pregnant Stroke-Prone Spontaneously Hypertensive rats.

**Conclusions:**

In summary, we demonstrate pregnancy-associated differences in urinary uromodulin: creatinine ratio and uromodulin excretion rate between chronic hypertensive and normotensive pregnancies. Further research is needed to fully understand uromodulin physiology in human pregnancy and establish uromodulin’s potential as a biomarker for renal adaptation and renal function in pregnancy.

**Graphical abstract:**

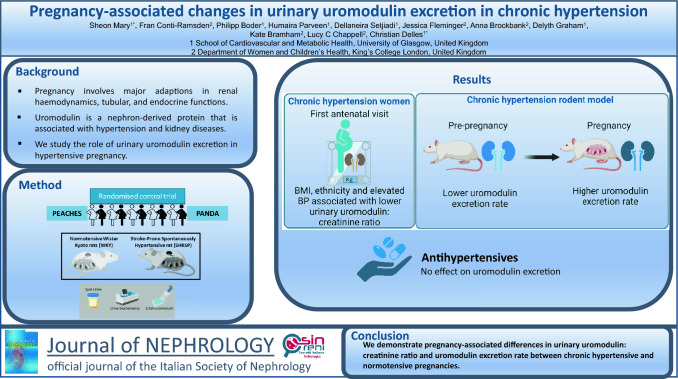

**Supplementary Information:**

The online version contains supplementary material available at 10.1007/s40620-023-01830-6.

## Introduction

Chronic hypertension affects 1–5% of pregnancies worldwide [1–3]. Compared to normotensive women, pregnant women with chronic hypertension have increased odds of adverse maternal, fetal and neonatal outcomes, including a high risk of superimposed pre-eclampsia (21–25%), preterm birth and stillbirth [4–6].

Pregnancy triggers a series of renal changes such as an increase in renal blood flow and glomerular filtration rate, and a decrease in serum creatinine, uric acid, blood urea nitrogen, sodium, and osmolality [7]. Chronic hypertension can affect renal adaptation in the early stages of pregnancy, and new onset hypertension in pregnancy causes further functional and structural changes in the kidney. Recent epidemiological studies suggest that hypertensive disorders of pregnancy affect renal function in the long term, with women with a history of pre-eclampsia and gestational hypertension having an increased long-term risk of chronic kidney disease (CKD) [8] and increased risks of cardiovascular, metabolic and cerebrovascular disease [4, 9, 10]. Biomarkers to identify women at highest risk to target postnatal follow-up and intervention are lacking. The renal physiological and pathological changes during pregnancy, particularly during pregnancies complicated by hypertension, are therefore of clinical significance and may have utility in kidney function assessment and prediction of long-term renal outcomes.

Uromodulin (also known as Tamm Horsfall protein) is exclusively synthesized by the epithelial cells lining the thick ascending limb of the loop of Henle and early distal convoluted tubule, and is the most abundant protein in a healthy individual’s urine [11–13]. Genetic studies have associated common variants of uromodulin with kidney function, risk of CKD and hypertension [14]. Furthermore, animal models and biochemical studies have shown that uromodulin plays an important role in the regulation of blood pressure (BP) and salt handling [15–17]. Urinary uromodulin excretion serves as a surrogate for tubular mass and function in the general population and in patients with renal diseases [18]. Several association studies have shown the prognostic value of uromodulin (urinary and plasma) in kidney function decline, incident CKD, and acute kidney injury (AKI), which are all well-summarised in recent reviews [11, 13].

Estimated glomerular filtration rate (eGFR) methods consistently underestimate renal function in pregnancy [19], and other filtration markers are inconsistent. Currently, serum creatinine is the only biomarker available to monitor maternal renal function during pregnancy. However, interpretation of variation in concentration is challenging due to dynamic changes in GFR with gestation, thus other biomarkers to identify acute kidney injury are needed. Increased levels of uromodulin peptides in urine have been proposed as a urinary peptidome marker of pre-eclampsia [20–22]. However, the role of uromodulin in chronic hypertensive pregnancy has not been well studied.

We examined the association between pre-existing hypertension and spot uromodulin concentration in a cohort of pregnant women. To further explore renal pathophysiology, we investigated uromodulin excretion and expression changes during pregnancy in a rodent model of chronic hypertension, i.e., the Stroke–Prone Spontaneously Hypertensive rat. Stroke-Prone Spontaneously Hypertensive rats are genetically predisposed to hypertension. They develop hypertension early in life and have sustained elevation of blood pressure during pregnancy [23]. Compared to Spontaneously Hypertensive rats, Stroke-Prone Spontaneously Hypertensive rats are more prone to renal damage [24]. Previous research, including our own, has identified Stroke-Prone Spontaneously Hypertensive rats as a suitable model for investigating chronic hypertension during pregnancy [23, 25–27]. Abnormal placentation leads to the release of antiangiogenic factors that may cause subclinical kidney injury [28]. We and others have shown abnormal placentation with release of antiangiogenic factors and kidney injury in Stroke-Prone Spontaneously Hypertensive rats [26, 29, 30].

## Materials and methods

### Human studies: experimental design

#### Cohort descriptions

Samples and data were obtained from women who participated in a randomised controlled trial of nifedipine versus labetalol for the treatment of chronic hypertension in pregnancy (PANDA trial, EudraCT Number: 2013-003144-23, International Standard Randomized Controlled Trials Number: 10.1186/ISRCTN40973936, Research Ethics Committee approval: 13/EE/0390) and an observational cohort study of women with hypertensive disorders of pregnancy (PEACHES study, Research Ethics Committee approval: 11/LO/1776).

Full details of the PANDA trial are reported elsewhere [31]. In brief, following informed consent, pregnant women aged 18 or over with a singleton pregnancy and a diagnosis of chronic hypertension before 20 weeks’ gestation requiring antihypertensive treatment prior to 27^+6^ weeks’ gestation were randomised to labetalol or nifedipine treatment at four obstetric units in the UK. Longitudinal spot urine samples were provided across pregnancy. Details of the PEACHES cohort have been published elsewhere [32]. In brief, pregnant women with chronic hypertension, pre-eclampsia (time of disease) and controls were prospectively enrolled between 20^+0^ and 36^+6^ weeks’ gestation from two centres. Spot urine samples were collected up to four times during pregnancy. In both studies urine samples were centrifuged at 1400 *g* for 10 min at 4 °C before storage at − 80 °C within 4 h.

In both studies, pregnancy and outcome data were recorded in dedicated electronic databases prospectively during pregnancy and following delivery by case note review by research midwives. Hypertensive disorder of pregnancy diagnoses were defined according to International Society for the Study of Hypertension guidelines [33].

#### Inclusion criteria

Samples from pregnant women with a diagnosis of chronic (pre-existing) hypertension, who did not develop superimposed pre-eclampsia (PANDA and PEACHES studies) and healthy controls (PEACHES study) with sufficient urine sample volumes for assay of uromodulin and creatinine concentrations were included in this study.

#### Uromodulin and biochemistry assays

Urinary uromodulin was quantified in spot urine samples using a human anti-uromodulin ELISA kit (DY5144-05, R&D systems, Abingdon, UK) as per the manufacturer’s guidelines. Urine samples were diluted with sample diluent buffer to fit the range of detection for uromodulin standards (62.5 to 4000 pg/mL). All samples were quantified on the same day to minimize variation. Plates were read at 450 nm in a microplate reader (Victor multilabel plate reader X3, Perkin Elmer Inc., Waltham, USA). Uromodulin concentrations were reported as urine uromodulin:creatinine (Umod:Crea) ratio. Creatinine concentrations were measured using Roche Cobas C311 Analyzer and commercially available human kits (Roche, Sussex, UK).

#### Statistical analysis

Plots of urinary creatinine concentration (g/mL) versus urinary uromodulin concentration (mg/mL) were visually inspected and a single outlier was removed. Urinary Umod:Crea ratio (mg/g) was calculated to account for varying urine concentrations. Standard distribution plots were used to assess the normality of data, and logarithmic transformation of Umod:Crea ratio was applied for formal testing of differences in view of a positively skewed distribution. Linear mixed models with random intercepts were used to account for repeated sampling episodes in some individuals, with participant ID set as random effects, and predictors of interest as fixed effects in models.

The following variables were tested for an association with urinary Umod:Crea ratio: Batch number, gestational age at the time of sampling, maternal age, maternal body mass index (BMI), maternal ethnicity, parity (binary primiparous/multiparous), smoking status, systolic and diastolic blood pressure at first antenatal visit (mean gestational age at first visit was 11.08 weeks (standard deviation 2.44 weeks), chronic hypertension diagnosis (binary variable), chronic kidney disease diagnosis (binary variable), and systolic and diastolic blood pressure closest to the time of sampling.

Change in urinary Umod:Crea (mg/g) ratio across pregnancy in participants with two or more sampling episodes was compared to change in antenatal systolic and diastolic blood pressure by selecting blood pressure measurements closest to the days of sampling.

All data analyses were performed in R version 4.1.3 [34]. The lme4 package was used to fit linear mixed models [35].

### Animal studies: experimental design

All procedures were performed as per Home Office regulation and with the United Kingdom Animals Scientific Procedures Act 1986 (PPL No. 70/9021) and ARRIVE Guidelines, and were approved by the institutional ethics review committee and performed at the University of Glasgow. Animals were housed under controlled environmental temperatures (21 ± 3 °C) and lighting (12-h light–dark cycles), maintained on a standard rat diet (rat no. 1 maintenance diet; Special Diet Services, Grangemouth, UK), and were provided tap water ad libitum.

Virgin female rats were time mated at 12 weeks (± 2 days) of age with males of respective strains. Gestational day 0.5 was confirmed by the presence of a copulation plug. Pregnant Stroke-Prone Spontaneously Hypertensive rats were randomly allotted to either placebo control (*n* = 8) or nifedipine (*n* = 7) or propranolol (*n* = 8) groups. Pre-pregnancy data were collected as a baseline at 11 weeks (± 2 days) of age for all the animals. Nifedipine (Sigma, Dorset, UK) was provided daily for three weeks at 10 mg/kg/day in 1 mL of baby food (Heinz Custard) and in-parallel, at 15 mg/kg/day in drinking water as previously established [26]. Propranolol (Sigma) 100 mg/kg/day in drinking water was given to rats daily for three weeks [36]. Systolic blood pressure was monitored weekly by tail-cuff plethysmography [37], in an operator-blinded fashion.

Animals were individually housed in metabolic cages at baseline and once every experimental week to estimate 24-h urine output. Urine samples were collected during these times. Animals were acclimatized 3 days before the first measurement. 24 h-urine samples were aliquoted and stored at − 80 °C.

Heparinized blood was collected by tail vein puncture (baseline and first two weeks of the experiment) under anaesthesia (isoflurane). At gestational day 18.5, heparinized and EDTA blood was collected by cardiac puncture and rats were euthanised by exsanguination under terminal general isoflurane anaesthesia. Biochemical plasma and urinary analyses for electrolyte, albumin and creatinine concentration were performed using Roche Cobas C311 Analyzer and commercially available rodent kits (Roche, Sussex, UK). Kidneys were dissected into two halves, snap-frozen in liquid nitrogen and stored at -80 °C until use.

#### Uromodulin quantitation by ELISA

Urinary and total kidney (lysate) uromodulin concentration was quantified using rat ELISA kit (Abcam, Cambridge, UK) as per the manufacturer’s guidelines for SimpleStep ELISA. The range of detection for uromodulin standards was 62.5 to 4000 pg/mL. Urine samples were diluted accordingly. For assessment of kidney uromodulin protein, the kidney was chopped into smaller pieces and washed in PBS before homogenization in the cell extraction buffer provided with the ELISA kit and using TissueLyser II (Qiagen, Manchester, UK). Lysate protein quantification was determined by Bradford assay (QuickStart Bradford protein assay, Bio-Rad Laboratories Ltd, Hertfordshire, UK) and an equal amount of protein was loaded for ELISA. To minimize variation, all sample protein extraction was performed on the same day. During the experiments, the analyst was blinded to sample details. For ELISA analysis, a four-parameter curve fit without constraints was used to determine the curve fit for standard values.

#### Quantitative real-time PCR

Isolation of total RNA from the kidney was performed using RNeasy mini spin kit (Qiagen, Manchester, UK) and the subsequent reverse transcription with High-Capacity RNA to cDNA kit (Thermo Fisher Scientific, Paisley, UK). Gene expression assay was performed using rat-specific TaqMan probes (Thermo Fisher Scientific, Paisley, UK): *Umod*, *Havcr1*(KIM-1), *Lcn2* (NGAL), and beta-actin* (Actb)* and TaqMan Fast Advanced Master Mix. Cycle threshold values obtained from QuantStudio software were manually used for calculation of delta cycle threshold and fold change.

#### Statistical analysis

Statistical analysis was performed on GraphPad Prism version 9. Student’s t-test or ANOVA or Welch test was performed as appropriate. The effect of two factors (*i.e.* strain and time of pregnancy) was tested by two-way ANOVA (or mixed model). Figure legends denote the type of test used for analysis. All statistical tests were 2-tailed and p-value < 0.05 was considered significant.

#### Terminology

The term Umod:Crea referred in the text represents the ratio of uromodulin concentration measured in spot urine to creatinine concentration, while uromodulin excretion rate represents uromodulin concentration measured in 24 h urine samples taking into account urine volume. Uromodulin excretion is used to refer to the general excretion process of this protein into urine.

## Results

### Maternal chronic hypertension is associated with lower urinary uromodulin

A total of 275 urine samples from 146 individuals were available for analysis. Women had a median of two spot urine samplings during pregnancy (range 1–5). A summary of participant demographics and pregnancy outcomes is shown in Table 1. Pre- and early-pregnancy renal function tests, and additional urine biochemistry results are available in Supplemental Table 1 and Supplemental Table 2, respectively. Women with chronic hypertension had normal early-pregnancy renal function which was comparable to that of controls (difference in serum creatinine: − 1.2 µmol/L, 95% CI − 17.6 to 15.1, p 0.884, adjusted for CKD status).

**Table 1:** Participant demographics and pregnancy outcomes in the whole study group and stratified by chronic hypertension (CHT) status

	All	Chronic hypertension	Controls
**Number of cases**	146	118	28
**Maternal age (years)**	34.64 (5.11)	35.27 (5.21)	32.00 (3.70)
**BMI** **category** **(kg/m**^**2**^**)**			
< 25	48 [33.1%]	26 [22.2%]	22 [78.6%]
25–29.9	37 [25.5%]	32 [27.4%]	5 [17.9%]
30–39.9	46 [31.7%]	45 [38.5%]	1 [3.6%]
40–50	14 [9.7%]	14 [12.0%]	0 [0.0%]
**Ethnic group**			
White	64 [43.8%]	45 [38.1%]	19 [67.9%]
Black	54 [37.0%]	50 [42.4%]	4 [14.3%]
East Asian	3 [2.1%]	3 [2.5%]	0 [0.0%]
South Asian	10 [6.8%]	9 [7.6%]	1 [3.6%]
Other	14 [9.6%]	10 [8.5%]	4 [14.3%]
Mixed	1 [0.7%]	1 [0.8%]	0 [0.0%]
**Multiparous**	83 [56.8%]	75 [63.6%]	8 [28.6%]
**Smoking status**			
Non-Smoker	138 [94.5%]	111 [94.1%]	27 [96.4%]
Stopped during pregnancy	5 [3.4%]	5 [4.2%]	0 [0.0%]
Smoker	3 [2.1%]	2 [1.7%]	1 [3.6%]
**Chronic hypertension**	118 [80.8%]	118 [100.0%]	0 [0.0%]
**Chronic kidney disease**	7 [4.8%]	7 [5.9%]	0 [0.0%]
**Diabetes (Type 1 or 2)**	0 [0.0%]	0 [0.0%]	0 [0.0%]
**Systolic BP at first visit (mmHg)**	129.2 (15.7)	133.3 (14.1)	111.7 (8.5)
**Diastolic BP at first visit (mmHg)**	83.0 (11.6)	86.3 (9.9)	68.8 (6.7)
**Pregnancy outcomess**			
**Birth outcome**			
Intrauterine death	2 [1.4%]	2 [1.7%]	0 [0.0%]
Live birth	138 [94.5%]	110 [93.2%]	28 [100.0%]
Termination of pregnancy	3 [2.1%]	3 [2.5%]	0 [0.0%]
Miscarriage	3 [2.1%]	3 [2.5%]	0 [0.0%]
**Infant sex**			
Female	69 [47.3%]	54 [45.8%]	15 [53.6%]
Male	75 [51.4%]	62 [52.5%]	13 [46.4%]
Missing	2 [1.4%]	2 [1.7%]	0 [0.0%]
**Birthweight (grams)**	2951(805)	2850 (824)	3373 (557)
**Preterm birth (< 37 weeks)**	28 [19.2]	26 [22.0]	2 [7.1]
**Preterm birth (< 34 weeks)**	13 [8.9]	13 [11.0]	0 [0.0]

Results are displayed as n [%] for categorical variables and mean (standard deviation) for continuous variables

**Table 2:** Univariable and multivariable adjusted linear mixed models of association between maternal variables and urinary uromodulin: creatinine ratio

	Univariable analysis	Multivariable analysis		
		Model 1* (BMI)	Model 2** (Ethnicity)	Model 3*** (BMI and Ethnicity)
**Chronic hypertension**	**0.69 (0.52–0.91)**	0.92 (0.66–1.29)	0.83 (0.62–1.12)	0.99 (0.72–1.37)
**BMI category (kg/m**^**2**^**)**				
< 25	Reference	–	Reference	–
25–29.9	**0.72 (0.55–0.95)**	–	**0.83 (0.63–1.10)**	–
30–39.9	**0.62 (0.48–0.80)**	–	**0.71 (0.54–0.92)**	–
40–50	**0.53 (0.37–0.77)**	–	**0.62 (0.43–0.91)**	–
**Ethnic group**				
Black	**0.59 (0.47–0.74)**	**0.65 (0.51–0.82)**	–	–
East Asian	0.61 (0.29–1.26)	0.58 (0.28–1.23)	–	–
Mixed	**0.15 (0.04–0.63)**	**0.21 (0.05–0.89)**	–	–
Other	0.88 (0.61–1.27)	0.83 (0.57–1.20)	–	–
South Asian	0.91 (0.61–1.35)	1.97 (0.65–1.45)	–	–
White	Reference	Reference	–	–
**Parity**				
**Multiparous**	**0.77 (0.62–0.96)**	0.84 (0.67–1.04)	0.97 (0.77–1.22)	0.98 (0.78–1.24)
**Systolic BP category (mmHg)**				
< 120	Reference	Reference	Reference	Reference
120–139	0.84 (0.64–1.10)	0.94 (0.72–1.24)	0.94 (0.72–1.23)	1.01 (0.77–1.32)
140–159	**0.69 (0.51–0.93)**	0.82 (0.60–1.13)	0.88 (0.64–1.19)	0.97 (0.71–1.33)
≥ 160	0.99 (0.56–1.77)	1.02 (0.57–1.82)	1.07 (0.61–1.89)	1.10 (0.63–1.92)

Results are displayed as ratios of the geometric mean with 95% confidence intervals of fixed effects (random effects not shown). Statistically significant results (*p* < 0.05) are shown in bold

*Multivariable model 1: Adjusted for maternal BMI

**Multivariable model 2: Adjusted for maternal ethnicity

***Multivariable model 3: Adjusted for maternal BMI and ethnicity

Chronic hypertension, increased maternal BMI (> 25 kg/m^2^), Black maternal ethnicity, multiparity and elevated systolic BP at the first antenatal visit were significantly associated with urinary Umod:Crea ratio in univariable analysis (Table 2, univariable analysis column). Samples from women with chronic hypertension had lower Umod:Crea ratios than controls (Geometric mean [95% CI]: 1.76 mg/g [1.59–1.93 mg/g] versus 2.54 mg/g [1.95–3.31 mg/g]). Samples from women with higher BMI had lower Umod:Crea ratios than those with a BMI in normal range, with Umod:Crea ratio reducing with increasing BMI (BMI < 25 kg/m^2^: 2.52 mg/g [2.13–2.97 mg/g], BMI 25–29.9: 1.87 mg/g [1.56–2.25], BMI 30–39.9 kg/m^2^: 1.56 mg/g [1.34–1.82 mg/g], BMI 40 + kg/m^2^: 1.32 mg/g [1.03–1.68 mg/g]). Samples from women of Black ethnic background had lower Umod:Crea ratios compared to White women (Black: 1.37 mg/g [1.21–1.55 mg/g], White: 2.34 mg/g [2.04–2.69 mg/g]). We used self-reported ethnicity, and only groups with sufficient statistical power were included in univariable analysis. Samples with women with elevated systolic BP (> 140 mmHg) at the first antenatal visit also had reduced Umod:Crea ratios although this was not consistent for women with severe hypertension (systolic BP > 160 mmHg), although there was a small number of samples in this group (*n* = 10). Sensitivity analysis excluding women with a past medical history of chronic kidney disease (*n* = 7) did not substantially alter results (data not shown).

In multivariable models following adjustment for maternal BMI or ethnicity, chronic hypertension, parity and systolic BP category at the first antenatal visit were no longer significantly associated with urinary Umod:Crea ratio (Table 2). Maternal BMI and Black ethnicity were associated with lower urinary Umod:Crea ratio independently of each other (Table 2).

**Fig. 1:**
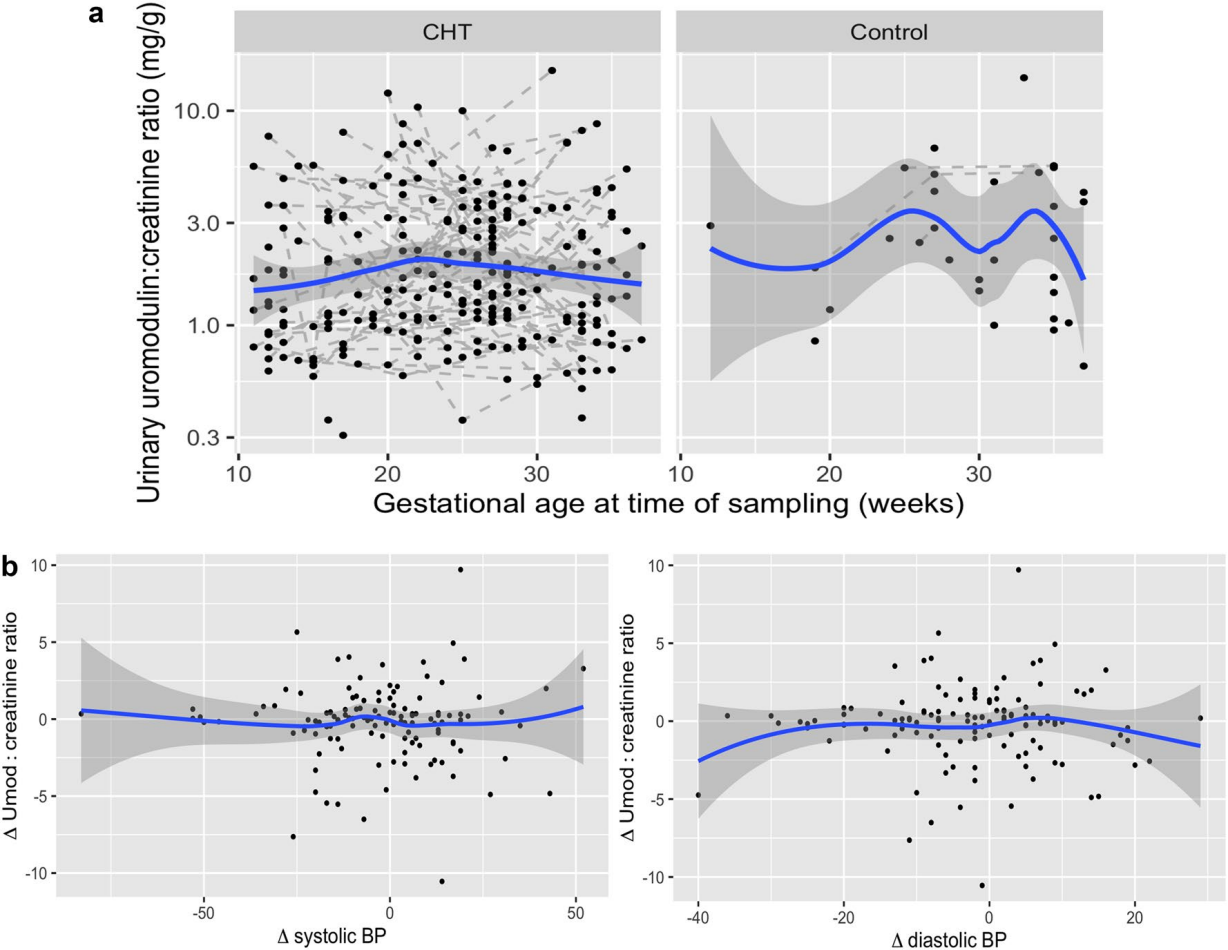
**a** Spot urinary uromodulin:creatinine ratio (mg/g) by gestational age (weeks) at sampling in participants with chronic hypertension (CHT) and controls. Repeated sampling episodes in a single participant are joined by solid lines. Trend lines with 95% confidence intervals were fitted using the Locally Estimated Scatterplot Smoothing (LOESS) method. No clear trend between urinary uromodulin:creatinine ratio and gestational age is observed in either CHT or controls. **b** Plot of change in urinary uromodulin:creatinine ratio (mg/g) and change in systolic and diastolic BP between sampling episodes in participants with repeated samples across pregnancy

In samples from women with chronic hypertension only, there was no difference in urinary Umod:Crea ratio for individuals prescribed nifedipine (*n* = 44) compared to labetalol monotherapy (*n* = 74) (ratio of geometric mean nifedipine v labetalol: 0.94 mg/g, 95% 0.74–1.21 mg/g). There was no association between gestational age at sampling and urinary Umod: Crea ratio (ratio of geometric mean in urinary Umod:Crea for 1 week increase in gestational age: 1.00 mg/g, 95% CI 0.99–1.02 mg/g) (Fig. 1a).

The median difference in days between the day of sampling and closest recorded BP was 1 day (IQR 0–3). There was no association between change in systolic and diastolic BP and change in urinary Umod:Crea ratio across samplings in participants (chronic hypertensives and controls) with repeated samples (Fig. 1b).

In summary, we observed a lower urinary Umod:Crea ratio in pregnant women with chronic hypertension compared to those without chronic hypertension, driven by maternal BMI and ethnicity with no trends observed in urinary Umod:Crea ratio across gestation in either the chronic hypertension or control groups.

### Pregnant Stroke-Prone spontaneously hypertensive rats as a model for chronic hypertensive pregnancy

**Fig. 2:**
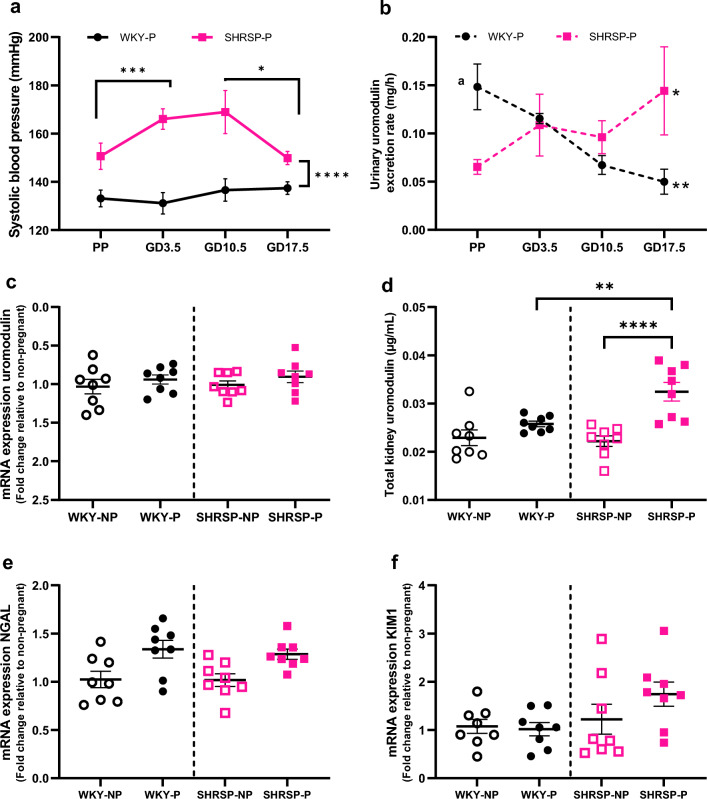
Uromodulin excretion rate increased pregnant Stroke-Prone Spontaneously Hypertensive rats **a** Systolic blood pressure measured by tail-cuff in Wistar Kyoto rats (WKY) (*n* = 8) and Stroke-Prone Spontaneously Hypertensive rats (SHRSP) (*n* = 8) before and during pregnancy. Repeated measure ANOVA with multiple comparisons. * *p* < 0.05, ***p < 0.001, ****p < 0.0001 strain difference in pregnant WKY and SHRSP. *GD* gestational day, *PP* pre-pregnancy. **b** The urinary uromodulin excretion in pregnant SHRSP increased compared to pregnant WKY. Repeated measure ANOVA with multiple comparisons. *p < 0.05 and **p < 0.01 difference compared to pre-pregnancy within the strain, a *p* < 0.05 pre-pregnancy strain difference. *GD* gestational day, *PP* pre-pregnancy. **c** There was no change in uromodulin mRNA level in pregnant (at GD 18.5) and age-matched non-pregnant female rats in both the strains (*n* = 8 per condition per strain). **d** Pregnant SHRSP (at GD 18.5) showed increase in total kidney uromodulin level compared to non-pregnant SHRSP and pregnant WKY. **p < 0.01, ****p < 0.0001 (Student’s *t* test). *NP* non-pregnan, *P* pregnant. The mRNA expression of **e** neutrophil gelatinase-associated lipocalin (NGAL) and **f** kidney injury marker-1 (KIM-1) were assessed in pregnant (at GD18.5) and age-matched non-pregnant female rats in both the strains. Student’s *t* test. *NP* Non-pregnant, *P* pregnant

Due to the absence of longitudinal urine uromodulin data in human pregnancy cohorts and the predominant use of spot urine samples in obstetric clinical practice, whilst 24-h urine samples are the gold standard for studying renal function, it has been challenging to study the relation between hypertension, pregnancy, and uromodulin in human populations. Additionally, there are no primary cultures of thick ascending limb cells or kidney biopsy samples from pregnant women available for such studies. As a result, we chose Stroke-Prone Spontaneously Hypertensive rats as the chronic hypertensive rodent model to further investigate the role of uromodulin in renal adaptation in pregnancy. The systolic blood pressure difference between Wistar Kyoto rats and Stroke-Prone Spontaneously Hypertensive rats pre-pregnancy was 17.5 ± 6.4 mmHg (p 0.016; repeated measure ANOVA for strain effect: *p* < 0.0001) (Fig. 2a). Pregnant Stroke-Prone Spontaneously Hypertensive rats showed an increase in systolic blood pressure of 15.4 ± 5.9 mmHg at gestational day 3.5, which was maintained throughout the second week, while a drop of 18.4 ± 6.6 mmHg was observed at gestational day17.5 (Fig. 2a). In contrast, pregnant Wistar Kyoto rats did not show changes in systolic blood pressure throughout pregnancy (Fig. 2a).

Kidney weight in non-pregnant Stroke-Prone Spontaneously Hypertensive rats was higher than in non-pregnant Wistar Kyoto rats (Supplemental Table 3). In pregnant animals, kidney weight normalised to maternal body weight was also significantly higher in Stroke-Prone Spontaneously Hypertensive rats than in Wistar Kyoto rats (mean difference: -0.55 ± 0.07 mg/g, *p* < 0.0001) (Supplemental Table 3). Pregnant Stroke-Prone Spontaneously Hypertensive rats were smaller in body weight compared to pregnant Wistar Kyoto rats (Supplemental Table 3). This difference was due to fetal number rather than fetal weight in pregnant Stroke-Prone Spontaneously Hypertensive rats (Supplemental Table 3). Other organs, such as the liver and spleen weighed less, while the heart weighed more in pregnant Stroke-Prone Spontaneously Hypertensive rats compared to pregnant Wistar Kyoto rats (Supplemental Table 3). Urinary and plasma biochemical parameters were measured once a week and are available in Supplemental Table 4.

### Pregnancy increases the expression and excretion rate of uromodulin in chronic hypertensive rodents

There was a significant difference in urinary uromodulin 24 h excretion rate between Wistar Kyoto rats and Stroke-Prone Spontaneously Hypertensive rats pre-pregnancy (0.083 ± 0.024 mg/h; *p* = 0.009) and during pregnancy (ANOVA mixed model, gestational day × strain interaction *p* = 0.002) (Fig. 2b). During pregnancy, in Wistar Kyoto rats there was a gradual decrease in urinary uromodulin (24 h excretion rate) from pre-pregnancy values (Fig. 2b). The fall in urinary uromodulin level in pregnant Wistar Kyoto rats can be observed as early as gestational day 3.5. In contrast, in Stroke-Prone Spontaneously Hypertensive rats there was a trend towards increased urinary uromodulin excretion during pregnancy (Fig. 2b).

At the mRNA level, there were no differences in uromodulin expression observed between non-pregnant and pregnant rats in both strains (Fig. 2c). However, there was a pregnancy-associated increased expression of uromodulin protein in total kidney extract in pregnant Stroke-Prone Spontaneously Hypertensive rats (FC: 1.5, *p* < 0.0001) and pregnant Wistar Kyoto rats (FC: 1.3, *p* = 0.005) compared to non-pregnant animals (Fig. 2d).

We investigated whether these changes in uromodulin were due to tubule injury in these rodents during pregnancy. As recognized markers for kidney injury, neutrophil gelatinase-associated lipocalin (NGAL) and Kidney Injury Molecule-1 (KIM-1) were included in our study to monitor renal health throughout gestation. The distal tubule injury marker NGAL showed a trend towards upregulation in both pregnant Wistar Kyoto rats and Stroke-Prone Spontaneously Hypertensive rats (FC: 1.3, p 0.06) compared to non-pregnant rats, however, there was no difference in expression between pregnant Wistar Kyoto rats and pregnant Stroke-Prone Spontaneously Hypertensive rats (Fig. 2e). The proximal tubule injury marker KIM-1 was upregulated only in pregnant Stroke-Prone Spontaneously Hypertensive rats (FC:1.6, *p* = 0.06) compared to pregnant Wistar Kyoto rats (Fig. 2f). The absence of significant kidney injury in these pregnant rats decreases the likelihood of uromodulin changes being attributed to kidney injury.

These finding demonstrate that the changes in uromodulin excretion rate and protein expression during pregnancy are opposite in Stroke-Prone Spontaneously Hypertensive rats and Wistar Kyoto rats and are likely due to post-transcriptional or post-translational modifications, and are not driven by tubular injury.

### Antihypertensives do not affect the uromodulin excretion rate

**Fig. 3:**
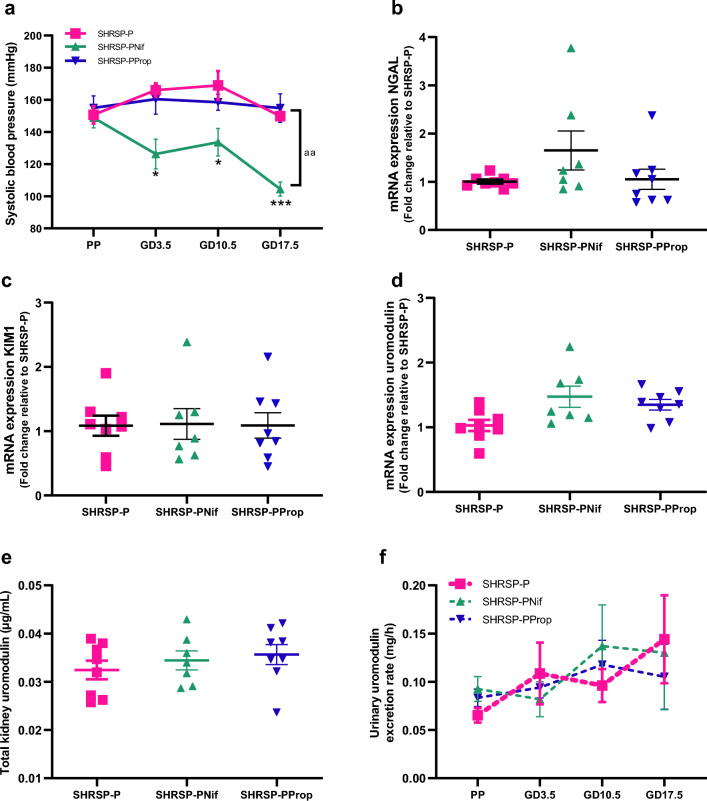
Effect of nifedipine and propranolol treatment on blood pressure and uromodulin. **a** Nifedipine (PNif, *n* = 7) reduced systolic blood pressure in pregnant SHRSP compared to propranolol (PProp, *n* = 8) and placebo pregnant SHRSP (*n* = 8). Repeated measure ANOVA with multiple comparisons. *p < 0.05 and ***p < 0.001 difference with nifedipine treatment in pregnant SHRSP, aa *p* < 0.01 difference between nifedipine- and placebo- treated pregnant SHRSP. Anti-hypertensive treatments in pregnant SHRSP did not reduce the kidney injury markers NGAL (**b**) and KIM1 (**c**). Student’s *T* test. Antihypertensive treatment did not change uromodulin in pregnant SHRSP at **d** mRNA level, **e** kidney protein level and **f** urinary excretion rate. Repeated measure ANOVA with multiple comparisons or Students’ *T* test. SHRSP-P: placebo treated pregnant SHRSP (*n* = 8); SHRSP-PNif: nifedipine treated pregnant SHRSP (*n* = 7) and SHRSP-PProp: propranolol treated pregnant SHRSP (*n* = 8)

We next investigated the role of blood pressure in pregnancy-associated uromodulin changes by treating pregnant Stroke–Prone Spontaneously Hypertensive rats with antihypertensives nifedipine (a calcium channel blocker) or propranolol (a beta-blocker). Nifedipine significantly reduced systolic blood pressure compared to placebo-treated pregnant Stroke-Prone Spontaneously Hypertensive rats (Fig. 3a). At the administered dose, there was variable response to propranolol treatment, however, the overall impact on blood pressure did not reach a statistically significant reduction (Fig. 3a and Supplemental Fig. 4).

Propranolol treatment increased the kidney weight in pregnant Stroke-Prone Spontaneously Hypertensive rats, while, nifedipine treatment did not (Supplemental Table 3). Nifedipine and propranolol treatment did not have any effect on NGAL and KIM-1 expression compared to pregnant Stroke-Prone Spontaneously Hypertensive rats (Figs. 3b, c).

Nifedipine- and propranolol-treated pregnant Stroke–Prone Spontaneously Hypertensive rats showed no change in kidney uromodulin mRNA expression (Fig. 3d) compared to placebo control pregnant Stroke–Prone Spontaneously Hypertensive rats. There was no effect on uromodulin protein expression (Fig. 3e). Both nifedipine and propranolol treatment did not significantly influence the excretion of urinary uromodulin compared to pregnant Stroke-Prone Spontaneously Hypertensive rats (Fig. 3f).

These finding demonstrate that neither nifedipine nor propranolol treatment have any significant effect on kidney uromodulin mRNA or protein expression, or on the excretion rate of urinary uromodulin in pregnant Stroke–Prone Spontaneously Hypertensive rats. This suggests that the difference in uromodulin excretion in pregnant hypertensive Stroke–Prone Spontaneously Hypertensive rats compared to control rats across pregnancy are not reversible with treatment of blood pressure during pregnancy.

## Discussion

The study revealed that maternal chronic hypertension, elevated BMI, Black maternal ethnicity, parity and high systolic BP during the first antenatal visit were individually associated with a lower urinary Umod:Crea ratio in human pregnancy. In adjusted models, maternal BMI and Black maternal ethnicity alone were independently associated with lower urinary Umod:Crea ratio. There is a complex relationship between ethnicity, BMI and pregnancy hypertension. Our findings suggest uromodulin excretion in pregnancy is most strongly driven by BMI and maternal ethnicity, however, investigation in a larger cohort is warranted.

To gain further insights into the role of uromodulin in chronic hypertensive pregnancy, a rodent model of chronic hypertension in pregnancy (the Stroke–Prone Spontaneously Hypertensive rats) was examined. In this model, we observed that the pre-pregnancy uromodulin excretion rates in chronic hypertensive rats (Stroke–Prone Spontaneously Hypertensive rats) are lower than in normotensive rats (Wistar Kyoto rats). Stroke–Prone Spontaneously Hypertensive rats had lower uromodulin excretion pre-pregnancy that gradually increased across gestation compared to normotensive Wistar Kyoto rats. This pattern of lower urinary uromodulin excretion rate pre-pregnancy and during the early stages of pregnancy observed in the Stroke-Prone Spontaneously Hypertensive model compared to Wistar Kyoto rodents was similar to the finding of lower Umod:Crea ratio in human chronic hypertensive pregnant women compared to normotensive controls.

In contrast to Stroke-Prone Spontaneously Hypertensive rats, normotensive (Wistar Kyoto) rodents had a reduction in uromodulin excretion across pregnancy. The pregnancy-associated changes in uromodulin levels in normotensive and hypertensive rodent pregnancy in opposite directions suggest multiple pregnancy factors regulating uromodulin excretion. We also showed that regulation is at the level of uromodulin translation and trafficking, possibly depending on the underlying pathophysiological conditions.

Furthermore, our findings suggest that the changes in uromodulin excretion during hypertensive compared to normotensive pregnancy are not reversed with control of blood pressure during pregnancy, as evidenced by comparisons between pregnant Stroke–Prone Spontaneously Hypertensive rats treated with antihypertensives versus controls. Antihypertensive agent class also did not affect uromodulin physiology in rodents. Neither nifedipine nor propanolol affected kidney uromodulin mRNA or protein expression or urinary excretion rate in rat models. In the human cohort, there were also no significant differences in Umod:Crea excretion observed between chronic hypertensive pregnant women prescribed nifedipine versus labetalol.

Despite a wealth of studies showing an association of uromodulin with various cardiovascular diseases and kidney diseases outside of pregnancy, there has been little investigation of the association between urinary, or serum uromodulin and pregnancy hypertension and its outcomes. Uromodulin in urine exists in two forms, namely polymerizing and nonpolymerizing uromodulin [38]. The function of nonpolymerizing uromodulin in urine is still unclear [38]. Whilst we were unable to assess polymerization status of uromodulin in this study, we have previously shown that nonpolymerizing uromodulin is increased in the urine of pregnant Stroke-Prone Spontaneously Hypertensive rats compared to pregnant Wistar Kyoto rats [39]. Increased uromodulin peptides from nonpolymerizing uromodulin have also been observed in urinary peptidomics data from women with pre-eclampsia compared to normotensive pregnant women [20, 21].

In keeping with our finding that BMI was strongly associated with Umod:Crea in pregnancy, serum uromodulin concentration is inversely correlated to BMI in non-pregnant adults with and without CKD [40]. Clinical studies have also shown that obesity is a risk factor for proteinuria, kidney damage and end-stage kidney disease [41–44]. Furthermore, BMI is known to be associated with altered renal haemodynamics [45–47], and BMI pre-pregnancy is known to be associated with elevated risk of developing hypertensive disorders during pregnancy [48].

To our knowledge, this is the first study to report that Black ethnicity is associated with lower urinary Umod:Crea ratio. Black ethnic background, in particular West African Ancestry, has also been found to be strongly associated with CKD and CKD progression [49, 50]. High risk APOL1 genetic variants are present in high frequency in individuals of West African descent and are likely to account for much of this increased risk [51, 52]. A retrospective cohort study using the USA National Inpatient Sample (from 2005 to 2015) also observed greater incidence of pregnancy-related acute kidney injury in women of Black and Hispanic ethnic backgrounds compared with White women, in addition to a greater risk of developing pre-eclampsia [53], which may reflect reduced nephron number that could be identified by lower Umod:Crea ratio.

It is striking that the strongest predictors of low urinary Umod:Crea ratio in our study, i.e., BMI and Black maternal ethnicity, are strongly associated with renal disease and hypertensive disorder of pregnancy risk. This suggests that further studies investigating the utility of uromodulin as a biomarker of renal function and adaptation in pregnancy and of long-term renal and cardiovascular health are warranted.

The lack of association between change in blood pressure and change in urinary Umod:Crea ratio in pregnancy in this study is not unexpected given that previous studies have reported conflicting results regarding uromodulin and blood pressure in adults, with both positive and negative associations reported [54, 55].

To our knowledge, this is the first study of uromodulin in chronic hypertensive human pregnancy. The strengths of the human study include diverse participant ethnic backgrounds and the multi-centre design. The limitations include a relatively small sample size, particularly of pregnant controls, precluding our ability to definitively determine normal physiological variations in Umod:Crea across pregnancy. In addition, samples were taken opportunistically rather than at specified gestational age windows, not all women had repeated measurements taken and non-pregnant controls were not included in the study. Furthermore, baseline renal mass and excretory renal function were not ascertained in pregnant women. In contrast to our experiments in rodents, we did not observe a change in the pattern of Umod:Crea with increase in gestational age in chronic hypertensive or control women. We speculate that this was because of the small sample size and opportunistic sampling rather than a longitudinal study design. Further research is required to determine physiological variation in urinary uromodulin concentration and excretion across pregnancy. In addition, lack of postnatal and long-term outcome data meant we were unable to assess the association between antenatal urinary Umod:Crea ratio and long-term renal outcomes.

Use of the Stroke–Prone Spontaneously Hypertensive rats strengthens our study as this chronic hypertension model does not require external factors such as salt, angiotensin, or surgical interventions to induce hypertension, renal injury or abnormal placentation. The pathway of uromodulin transcription, post-translational secretion from thick ascending limb cells and release into urine during pregnancy can only be dissected through animal models. It should be noted that some of the chronic hypertensive women in this study were on anti-hypertensive medications prior to their pregnancy, while in case of rodent study, antihypertensive treatment began after gestational day 0.5. However, similar to the human cohort, we did not observe any changes in uromodulin excretion with these anti-hypertensive treatments. Currently, as there are no pharmacological agents capable of regulating uromodulin expression, we were unable to further explore its mechanisms and significance in this model.

Lower urinary and serum uromodulin concentration levels are an early indicator of poor tubular function [56], early stages of CKD [40] and risk of acute kidney injury in non-pregnant adults [57]. The vast majority of women in our study with chronic hypertension had normal kidney function at the beginning of pregnancy as assessed by serum creatinine. Currently, there are no tests available to assess renal adaptation in pregnancy, reduced renal reserve due to low nephron number, susceptibility to AKI in pregnancy, or biomarkers for postpartum risk stratification. Whilst this study suggests urinary uromodulin may have utility in clinical practice in hypertensive pregnancy, future research is needed to determine to what extent uromodulin can be used as a clinical biomarker for renal adaptation and renal function during pregnancy and post-partum CKD risk in high-risk women. This study is also the first to report an association between maternal ethnicity and urinary uromodulin. This should also be confirmed in larger studies and may provide mechanistic insights into CKD risk across ethnic groups.

In summary, we observe that chronic hypertension influences urinary uromodulin concentration and excretion during pregnancy. Whether urinary uromodulin may be a biomarker of renal adaptation in pregnancy and long-term CKD risk in hypertensive pregnancy warrants further research.

### Supplementary Information

Below is the link to the electronic supplementary material.

Supplementary file1 (DOCX 53 kb)

## Data Availability

The authors confirm that the data supporting the findings of this study are available within the article and its supplementary materials. Any other data supporting this study will be available on request from the corresponding authors [SM].
