# Supplementary material for: Pregnancy-associated changes in urinary uromodulin excretion in chronic hypertension
Source: J Nephrol. 2024 Jan 18;37(3):597–610. doi: 10.1007/s40620-023-01830-6 (PMC11150301; doi:10.1007/s40620-023-01830-6)
Supplement: Supplementary file 1 — Supplementary file1 (DOCX 53 kb) [file 40620_2023_1830_MOESM1_ESM.docx]

**Pregnancy-associated changes in urinary uromodulin excretion in chronic hypertension**

Sheon Mary^1*#^, Fran Conti-Ramsden^2#^, Philipp Boder^1^, Humaira Parveen^1^, Dellaneira Setjiadi^1^, Jess Fleminger^2^, Anna Brockbank^2^, Delyth Graham^1^, Kate Bramham^2^, Lucy C Chappell^2^, Christian Delles^1*^

School of Cardiovascular and Metabolic Health, University of Glasgow, United Kingdom

Department of Women and Children’s Health, King’s College London, United Kingdom

**Corresponding author:**

Prof. Christian Delles

School of Cardiovascular and Metabolic Health, University of Glasgow, BHF Glasgow Cardiovascular Research Centre, 126 University Place, Glasgow G12 8TA

Email: [Christian.Delles@glasgow.ac.uk](mailto:Christian.Delles@glasgow.ac.uk)

Dr Sheon Mary

School of Cardiovascular and Metabolic Health, University of Glasgow, BHF Glasgow Cardiovascular Research Centre, 126 University Place, Glasgow G12 8TA

Email : [Sheon.Samji@glasgow.ac.uk](mailto:Sheon.Samji@glasgow.ac.uk)

**#**shared first authors

**Short title:** Uromodulin in pregnancy.

**Table of Contents:**

Supplemental Table 1: Pre-pregnancy and early pregnancy renal function test results in the study cohort stratified by chronic hypertension and chronic kidney disease status.

Supplemental Table 2: Urine biochemistry in the study cohort.

Supplemental Table 3: Maternal tissue weight in animals

Supplemental Table 4: Urine and plasma biochemical parameters in animals.

Supplemental Figure 4: Pregnant SHRSP treated with 100mg/kg/day of propranolol.

**Supplementary Table 1**: Pre-pregnancy and early pregnancy renal function test results in study cohort stratified by chronic hypertension and chronic kidney disease status. *Results are reported as n (%) or median [interquartile range] as appropriate.*

|  | **Chronic hypertension**  **(n = 118)** | | **Controls**  **(n = 28)** |
| --- | --- | --- | --- |
|  | **No CKD (n = 111)** | **CKD (n = 7)** |  |
| **PRE-PREGNANCY** |  |  |  |
| *Number of individuals with pre-pregnancy eGFR record* | 4 (3.6%) | 0 (0.0%) | 1 (3.6%) |
| *eGFR where recorded (mL/min/m^2^)* | 101 [72-106] | - | 105 [NA] |
| **EARLY PREGNANCY** |  |  |  |
| *Number of individuals with booking creatinine record* | 70 (63.1%) | 7 (100.0%) | 6 (21.4%) |
| *Serum creatinine where recorded (*µmol/L*)* | 53 [9 – 80] | 99 [61-216] | 53 [40-78] |
| *No. individuals with booking protein:creatinine ratio record* | 38 (34.2%) | 3 (42.9%) | 2 (7.1%) |
| *Urinary where recorded (mg/mmol)* | 8 [3-40] | 174 [41-691] | 5.5 [0-11] |

*CKD = chronic kidney disease, eGFR = estimated glomerular filtration rate, PCR = protein creatinine ratio.*

**Supplemental Table 2:** Urine biochemistry in study cohort. *Results are presented as median [interquartile range].*

| **Urine parameters** | **Overall** | **CHT** | **Control** | **P value** |
| --- | --- | --- | --- | --- |
| n | 275 | 243 | 32 |  |
| Albumin (mg/L) | 5.90 [1.60, 17.25] | 7.00 [1.75, 18.25] | 1.80 [1.20, 3.55] | 0.001 |
| Urea (mmol/L) | 198.80 [103.25, 285.00] | 205.60 [121.95, 295.30] | 116.65 [75.00, 224.88] | 0.002 |
| Calcium (mmol/L) | 2.79 [1.42, 5.20] | 2.75 [1.38, 5.20] | 2.99 [1.69, 5.42] | 0.9 |
| Magnesium (mmol/L) | 2.14 [1.16, 3.48] | 2.20 [1.18, 3.48] | 1.68 [1.12, 3.38] | 0.307 |
| Phosphate (mmol/L) | 11.60 [6.40, 21.10] | 13.00 [6.80, 21.20] | 6.60 [3.65, 12.60] | 0.003 |
| Creatinine (µmol/L) | 8360.00 [4133.00, 13713.00] | 8929.00 [4618.00, 14549.50] | 4104.50 [2368.50, 7745.50] | <0.001 |
| Sodium (mmol/L) | 75.00 [39.00, 118.50] | 80.00 [40.00, 120.50] | 59.00 [34.00, 101.25] | 0.293 |
| Potassium (mmol/L) | 53.84 [30.71, 78.07] | 57.16 [32.52, 80.76] | 36.00 [27.21, 54.72] | 0.003 |
| Chloride (mmol/L) | 87.50 [47.25, 134.20] | 90.90 [49.55, 135.25] | 72.20 [36.33, 110.15] | 0.083 |

**Supplemental Table 3:** Maternal tissue weight in animals

|  | WKY-NP | WKY-P | SHRSP-NP | SHRSP-P | SHRSP-PCC | SHRSP-PBB |
| --- | --- | --- | --- | --- | --- | --- |
| Body weight (BW, g) | 212.8 ± 5.0 | 269.8 ± 6.8^aaaa^ | 185.6 ± 3.3^aaa^ | 241.4 ± 5.7^bbb,cccc^ | 236.9 ± 2.7 | 222.3 ± 4.6^dd,e^ |
| Heart (mg) | 751.5 ± 18.9 | 796.7 ± 15.6 | 773.5 ± 19.5 | 842.5 ± 16.3^cc^ | 824.5 ± 21.4 | 810.9 ± 15.0 |
| Heart (mg/g BW) | 3.5 ± 0.0 | 3.0 ± 0.1^aaaa^ | 4.2 ± 0.1^aaaa^ | 3.5 ± 0.1^bbb,cccc^ | 3.5 ± 0.1 | 3.7 ± 0.1 |
| Kidney (mg) | 615.1 ± 16.4 | 625.9 ± 14.0 | 659.5 ± 17.0 | 695.2 ± 15.8^bb^ | 715.8 ± 23.6 | 753.2 ± 10.1^d^ |
| Kidney (mg/g BW) | 2.9 ± 0.0 | 2.3 ± 0.1^aaaa^ | 3.6 ± 0.1^aaaa^ | 2.9 ± 0.1^bbbb,cccc^ | 3.0 ± 0.1 | 3.4 ± 0.1^dddd,ee^ |
| Lung (mg) | 1,552.0 ± 45.4 | 1,619.0 ± 40.7 | 1,371.0 ± 57.5^a^ | 1,379.0 ± 57.6^bb^ | 1,437.0 ± 72.4 | 1,268.0 ± 50.9^e^ |
| Lung (mg/ g BW) | 7.3 ± 0.1 | 6.0 ± 0.1^aaa^ | 7.4 ± 0.2 | 5.7 ± 0.3^cccc^ | 6.1 ± 0.3 | 5.7 ± 0.3 |
| Spleen (mg) | 569.4 ± 22.3 | 675.8 ± 37.3^aaa^ | 416.1 ± 13.0^aaaa^ | 447.4 ± 9.2^bbbb^ | 461.8 ± 12.8 | 443.1 ± 13.2 |
| Spleen (mg/ g BW) | 2.7 ± 0.1 | 2.5 ± 0.1 | 2.2 ± 0.0^aaaa^ | 1.9 ± 0.1^bbbb,ccc^ | 1.9 ± 0.1 | 2.0 ± 0.1 |
| Liver (mg) | 9,318.0 ± 249.3 | 13,597.0 ± 284.0^aaaa^ | 7,419.0 ± 180.4^aaaa^ | 11,204.0 ± 274.0^bbbb,cccc^ | 11,350.0 ± 190.3 | 10,095.0 ± 229.2^dd,eee^ |
| Liver (mg/ g BW) | 43.8 ± 0.8 | 50.5 ± 1.2^aaaa^ | 40.0 ± 0.5^aa^ | 46.4 ± 0.5^bb,cccc^ | 47.9 ± 0.5 | 45.5 ± 1.1 |
| Fetal body weight (mg) |  | 907.8 ± 40.9 |  | 1,294.0 ± 81.0^bbb^ | 1,382.0 ± 84.5 | 1,044.0 ± 84.7^d,ee^ |
| Fetal head weight (mg) |  | 319.6 ± 10.7 |  | 423.7 ± 21.9^bbb^ | 444.2 ± 20.6 | 365.0 ± 22.6^d,ee^ |
| No. of Fetus |  | 14 ± 1 |  | 10 ± 1^b^ | 10 ± 1 | 11 ± 1 |

Values represent Mean ± SEM, NP: non-pregnant, P: pregnant, CC: nifedipine and BB: propranolol.

^a^ comparison to WKY-NP, ^b^ comparison to WKY-P, ^c^ compared to SHRSP-NP, ^d^ compared to SHRSP-P, ^e^ compared to SHRSP-PCC

**Supplemental Table 4:** Urine and plasma biochemical parameters in animals.

|  | Gestational week | WKY-P  (n=8) | SHRSP-P  (n=8) | SHRSP-PCC  (n=7) | SHRSP-PBB  (n=8) |
| --- | --- | --- | --- | --- | --- |
| *Urine parameters* | | | | | |
| Sodium (mmol/day) | PP | 0.45 ± 0.07 | 0.44 ± 0.10 | 0.40 ± 0.08 | 0.73 ± 0.13 |
|  | GW1 | 0.75 ± 0.14^x^ | 0.71 ± 0.14 | 0.60 ± 0.18 | 1.08 ± 0.15^xx^ |
|  | GW2 | 0.73 ± 0.25 | 0.75 ± 0.12 | 1.10 ± 0.28^x^ | 1.33 ± 0.26^x^ |
|  | GW3 | 0.76 ± 0.19 | 0.39 ± 0.05 | 0.83 ± 0.19 | 0.91 ± 0.23 |
| Potassium (mmol/day) | PP | 1.63 ± 0.08 | 1.38 ± 0.08 | 1.69 ± 0.11 | 1.46 ± 0.09 |
|  | GW1 | 2.25 ± 0.06^xx^ | 2.14 ± 0.09^xxx^ | 1.51 ± 0.18^a^ | 1.56 ± 0.08^aaaa,bb^ |
|  | GW2 | 2.51 ± 0.17^xx^ | 2.37 ± 0.17^xxx^ | 2.19 ± 0.13 | 2.01 ± 0.15^x^ |
|  | GW3 | 2.75 ± 0.08^xxx,y^ | 2.17 ± 0.12^aa,xx^ | 2.12 ± 0.21 | 1.54 ± 0.19^aaa^ |
| Calcium (mmol/day) | PP | 0.05 ± 0.01 | 0.06 ± 0.01 | 0.07 ± 0.01 | 0.07 ± 0.01 |
|  | GW1 | 0.14 ± 0.02^xx^ | 0.14 ± 0.01^xxx^ | 0.08 ± 0.02^xx^ | 0.07 ± 0.01^a,bb^ |
|  | GW2 | 0.13 ± 0.04 | 0.14 ± 0.02^xx^ | 0.13 ± 0.01^x^ | 0.07 ± 0.01^b,cc^ |
|  | GW3 | 0.23 ± 0.02^xx,z^ | 0.16 ± 0.01^xxx^ | 0.17 ± 0.02^yy^ | 0.05 ± 0.01^aaa,bbb,cc^ |
| Magnesium (mmol/day) | PP | 0.22 ± 0.01 | 0.16 ± 0.01 | 0.22 ± 0.02 | 0.15 ± 0.01^a,c^ |
|  | GW1 | 0.32 ± 0.02^x^ | 0.32 ± 0.01^xxx^ | 0.14 ± 0.02^aaa,bbb,x^ | 0.10 ± 0.01^aaaa,bbbb,x^ |
|  | GW2 | 0.33 ± 0.06 | 0.31 ± 0.02^xx^ | 0.23 ± 0.02^y^ | 0.11 ± 0.01^a,bbb,cc^ |
|  | GW3 | 0.40 ± 0.03^xxx^ | 0.24 ± 0.02^aa,xx^ | 0.24 ± 0.03^aa,yy^ | 0.09 ± 0.01^aaaa,bbb,cc,x^ |
| Phosphate (mmol/day) | PP | 0.02 ± 0.01 | 0.08 ± 0.02 | 0.18 ± 0.04^a^ | 0.04 ± 0.02 |
|  | GW1 | 0.03 ± 0.02 | 0.11 ± 0.03 | 0.13 ± 0.04 | 0.08 ± 0.03 |
|  | GW2 | 0.09 ± 0.05 | 0.13 ± 0.04 | 0.08 ± 0.02 | 0.08 ± 0.03 |
|  | GW3 | 0.12 ± 0.04 | 0.22 ± 0.04^x^ | 0.14 ± 0.05 | 0.11 ± 0.02 |
| Chloride (mmol/day) | PP | 1.20 ± 0.10 | 1.04 ± 0.10 | 1.07 ± 0.12 | 1.33 ± 0.15 |
|  | GW1 | 1.73 ± 0.21^x^ | 1.54 ± 0.16^x^ | 1.21 ± 0.26 | 1.88 ± 0.21^x^ |
|  | GW2 | 1.65 ± 0.26 | 1.67 ± 0.18 | 1.95 ± 0.41 | 2.44 ± 0.35^x^ |
|  | GW3 | 1.77 ± 0.18^xx^ | 1.29 ± 0.10 | 1.57 ± 0.28 | 1.90 ± 0.34 |
| Creatinine (µmol/day) | PP | 54.15 ± 2.82 | 41.58 ± 2.44 | 50.91 ± 1.08^b^ | 43.59 ± 1.78^a,c^ |
|  | GW1 | 66.05 ± 2.20^x^ | 55.13 ± 1.84^aa,xx^ | 51.66 ± 4.54 | 48.58 ± 1.78^aaa^ |
|  | GW2 | 72.10 ± 3.45^xxx^ | 62.93 ± 2.72^xx^ | 57.27 ± 2.64^a^ | 50.47 ± 2.76^aa,b,x^ |
|  | GW3 | 75.81 ± 2.33^xx,y^ | 60.52 ± 3.33^a,xx^ | 57.54 ± 2.35^aaa^ | 45.04 ± 3.32^aaaa,b,c^ |
| Urea (mmol/day) | PP | 5.19 ± 0.39 | 5.09 ± 0.31 | 6.26 ± 0.30 | 5.07 ± 0.28 |
|  | GW1 | 5.94 ± 0.26 | 6.77 ± 0.39^xx^ | 5.52 ± 0.49 | 5.51 ± 0.37 |
|  | GW2 | 6.65 ± 0.50^xx^ | 7.89 ± 0.30^xx^ | 7.14 ± 0.46 | 6.65 ± 0.38^xx^ |
|  | GW3 | 8.24 ± 0.43^xx,yy^ | 7.45 ± 0.43^x^ | 6.83 ± 0.37 | 5.57 ± 0.54^aa^ |
| *Plasma parameters* | | | | | |
| Sodium (mmol/day) | GW3 | 135.38 ± 0.38 | 134.75 ± 0.31 | 135.86 ± 0.88 | 131.25 ± 1.19^aa,b,cc^ |
| Potassium (mmol/day) | PP | 6.60 ± 0.37 | 6.01 ± 0.45 | 5.52 ± 0.32 | 5.40 ± 0.14 |
|  | GW1 | 6.58 ± 0.22 | 5.87 ± 0.29 | 6.54 ± 0.34 | 7.49 ± 0.40^b^ |
|  | GW2 | 6.38 ± 0.20 | 6.53 ± 0.63 | 5.46 ± 0.40 | 7.26 ± 0.36^c^ |
|  | GW3 | 4.49 ± 0.20^x,yy,zz^ | 4.78 ± 0.45 | 4.75 ± 0.17^yy^ | 5.31 ± 0.31^yy,z^ |
| Calcium (mmol/day) | PP | 2.55 ± 0.04 | 2.65 ± 0.03 | 2.63 ± 0.03 | 2.59 ± 0.03 |
|  | GW1 | 2.53 ± 0.04 | 2.62 ± 0.03 | 2.51 ± 0.02 | 2.59 ± 0.02 |
|  | GW2 | 2.51 ± 0.05 | 2.59 ± 0.05 | 2.53 ± 0.04 | 2.53 ± 0.03 |
|  | GW3 | 2.72 ± 0.03^x,yy,z^ | 2.74 ± 0.02 | 2.79 ± 0.08^z^ | 2.78 ± 0.03^yy,zzzz^ |
| Magnesium (mmol/day) | PP | 0.96 ± 0.07 | 0.88 ± 0.02 | 0.86 ± 0.04 | 0.85 ± 0.02 |
|  | GW1 | 0.96 ± 0.04 | 0.91 ± 0.03 | 0.81 ± 0.02^a^ | 0.86 ± 0.02 |
|  | GW2 | 0.95 ± 0.04 | 0.87 ± 0.04 | 0.79 ± 0.02^a^ | 0.84 ± 0.03 |
|  | GW3 | 0.77 ± 0.01^yy,z^ | 0.86 ± 0.02^aa^ | 0.84 ± 0.02^a^ | 0.84 ± 0.02^a^ |
| Phosphate (mmol/day) | PP | 1.64 ± 0.13 | 1.27 ± 0.13 | 1.34 ± 0.16 | 1.37 ± 0.13 |
|  | GW1 | 1.76 ± 0.16 | 1.58 ± 0.14 | 1.58 ± 0.16 | 1.80 ± 0.13 |
|  | GW2 | 2.11 ± 0.13 | 1.78 ± 0.07^x^ | 1.54 ± 0.09^a^ | 1.78 ± 0.06 |
|  | GW3 | 1.70 ± 0.07 | 1.39 ± 0.08 | 1.27 ± 0.14 | 1.65 ± 0.08 |
| Chloride (mmol/day) | GW3 | 85.33 ± 0.66 | 85.44 ± 0.26 | 85.04 ± 0.92 | 88.15 ± 1.21 |
| Creatinine (µmol/day) | PP | 57.38 ± 8.71 | 28.13 ± 2.90^a^ | 30.33 ± 5.32 | 32.60 ± 3.41 |
|  | GW1 | 65.00 ± 8.21 | 33.71 ± 2.19^a^ | 28.86 ± 3.63^a^ | 37.25 ± 3.68^a^ |
|  | GW2 | 51.13 ± 5.52 | 31.88 ± 2.73^a^ | 26.86 ± 3.59^a^ | 31.50 ± 3.15^a^ |
|  | GW3 | 30.25 ± 1.70^y,z^ | 25.25 ± 2.08^y^ | 24.57 ± 1.04 | 22.38 ± 1.00^aa,y^ |
| Urea (mmol/day) | PP | 6.83 ± 0.79 | 5.98 ± 0.17 | 5.22 ± 0.49 | 4.58 ± 0.36^b^ |
|  | GW1 | 6.43 ± 0.41 | 6.14 ± 0.68 | 5.11 ± 0.64 | 5.60 ± 0.29 |
|  | GW2 | 6.75 ± 0.63 | 5.74 ± 0.54 | 4.96 ± 0.56 | 5.86 ± 0.29 |
|  | GW3 | 5.45 ± 0.13 | 5.98 ± 0.28 | 6.26 ± 0.34 | 6.66 ± 0.38 |
| Packed cell volume (RBC)% | PP | 41.62 ± 0.50 | 44.94 ± 0.78^a^ | 45.87 ± 0.79^aa^ | 44.29 ± 0.95 |
|  | GW1 | 39.70 ± 0.67 | 44.26 ± 0.62^aaa^ | 41.00 ± 1.72 | 45.86 ± 0.79^aaa^ |
|  | GW2 | 35.37 ± 1.26^xx^ | 39.34 ± 1.18^xx,y^ | 40.40 ± 0.92^aa,x^ | 39.26 ± 1.30^x,yy^ |
|  | GW3 | 31.08 ± 1.90^xx,y^ | 40.61 ± 1.77^a^ | 38.38 ± 0.81^a,x^ | 39.78 ± 1.03^aa,x,yy^ |

Mean +/- SEM

One-way ANOVA for single time points. 2-way ANOVA repeated measure with Tukey’s multiple comparison test: a,b,c,x,y,z, p<0.05; aa,bb,cc,xx,yy,zz, p<0.01; aaa,bbb,ccc,xxx,yyy,zzz, p<0.001

a: Pregnant(P) WKY *versus* SHRSP (pregnant, on nifedipine (CC) or propranolol (BB))

b: Pregnant SHRSP *versus* pregnant SHRSP on Nifedipine or Propranolol

c: Pregnant SHRSP on nifedipine *versus* pregnant SHRSP on Propranolol

x: pre-pregnancy *versus* gestational week 1or 2 or3

y: gestational week 1 *versus* gestational week 2or 3

z: gestational week 2 *versus* gestational week 3


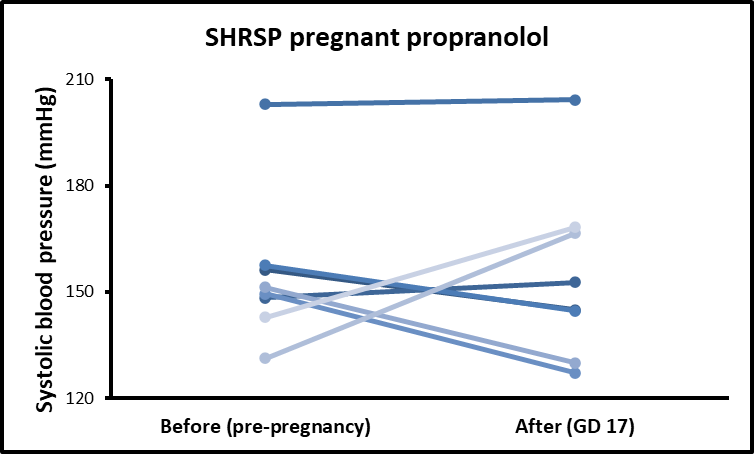
**Supplemental Figure 4: Pregnant SHRSP treated with 100mg/kg/day of propranolol.** Individualized analysis of the data for the systolic blood pressure response to propranolol in pregnant SHRSP as depicted in the figure below, reveals a diverse range of outcomes. Specifically, among the rats treated with propranolol, three displayed decreased blood pressure, two exhibited no discernible changes, and two demonstrated an unresponsive increase in blood pressure. The dose of propranolol (100mg/kg/day) was administered through drinking water, following the protocol outlined in a prior reference (PMID: 693116 or ref [36] in text) involving pregnant WKY rats. A dose exceeding 150mg/kg/day lead to abnormal weight loss in both pregnant rats and neonates [36]. In our study involving pregnant SHRSP treated with 100mg/kg/day of propranolol, we noted an average maternal weight loss of approximately 20g and fetal weight loss of around 250mg when compared to pregnant SHRSP (Supplemental Table 3).
